# 

**Published:** 1795

**Authors:** 


### MEDICAL FACTS

AND

### OBSERVATIONS.

VOL. VI.

